# Assembly of Replication-Incompetent African Horse Sickness Virus Particles: Rational Design of Vaccines for All Serotypes

**DOI:** 10.1128/JVI.00548-16

**Published:** 2016-07-27

**Authors:** Valeria Lulla, Aleksei Lulla, Kerstin Wernike, Andrea Aebischer, Martin Beer, Polly Roy

**Affiliations:** a Department of Pathogen Molecular Biology, Faculty of Infectious and Tropical Diseases, London School of Hygiene and Tropical Medicine, London, United Kingdom; b Institute of Diagnostic Virology, Friedrich-Loeffler Institut, Greifswald, Germany; Wake Forest University

## Abstract

African horse sickness virus (AHSV), an orbivirus in the Reoviridae family with nine different serotypes, causes devastating disease in equids. The virion particle is composed of seven proteins organized in three concentric layers, an outer layer made of VP2 and VP5, a middle layer made of VP7, and inner layer made of VP3 that encloses a replicase complex of VP1, VP4, and VP6 and a genome of 10 double-stranded RNA segments. In this study, we sought to develop highly efficacious candidate vaccines against all AHSV serotypes, taking into account not only immunogenic and safety properties but also virus productivity and stability parameters, which are essential criteria for vaccine candidates. To achieve this goal, we first established a highly efficient reverse genetics (RG) system for AHSV serotype 1 (AHSV1) and, subsequently, a VP6-defective AHSV1 strain in combination with in *trans* complementation of VP6. This was then used to generate defective particles of all nine serotypes, which required the exchange of two to five RNA segments to achieve equivalent titers of particles. All reassortant-defective viruses could be amplified and propagated to high titers in cells complemented with VP6 but were totally incompetent in any other cells. Furthermore, these replication-incompetent AHSV particles were demonstrated to be highly protective against homologous virulent virus challenges in type I interferon receptor (IFNAR)-knockout mice. Thus, these defective viruses have the potential to be used for the development of safe and stable vaccine candidates. The RG system also provides a powerful tool for the study of the role of individual AHSV proteins in virus assembly, morphogenesis, and pathogenesis.

**IMPORTANCE** African horse sickness virus is transmitted by biting midges and causes African horse sickness in equids, with mortality reaching up to 95% in naive horses. Therefore, the development of efficient vaccines is extremely important due to major economic losses in the equine industry. Through the establishment of a highly efficient RG system, replication-deficient viruses of all nine AHSV serotypes were generated. These defective viruses achieved high titers in a cell line complemented with VP6 but failed to propagate in wild-type mammalian or insect cells. Importantly, these candidate vaccine strains showed strong protective efficacy against AHSV infection in an IFNAR^−/−^ mouse model.

## INTRODUCTION

Among closely related orbiviruses (Reoviridae family), such as bluetongue virus (BTV), epizootic hemorrhagic disease virus (EHDV), and African horse sickness (AHS) virus (AHSV), AHSV is known to cause the most severe morbidity and mortality in infected animals. In susceptible horses, AHSV can cause different forms of disease ranging from mild fever to an acute form, characterized by high fever, depression, respiratory symptoms, severe weight loss, rough hair, lethargy, and apathy, and mortality reaches up to 95% (1). Although AHS is endemic to sub-Saharan Africa, occasional outbreaks which had significant social and economic impacts were reported in North Africa, Pakistan, India, Spain, and Portugal. These three orbiviruses are transmitted by midges of Culicoides species, which circulate throughout Europe and the United States, thus increasing the geography of the potential risk of an AHSV outbreak, similar to the risk from BTV and EHDV (2). The genome sequence of AHSV is sufficiently divergent from the genome sequences of BTV and EHDV (3), and unlike BTV, which has 27 serotypes, only 9 serotypes of AHSV (AHSV serotype 1 [AHSV1] to AHSV9) have been distinguished to date. Vaccination with a polyvalent live-attenuated vaccine (LAV) against AHSV is currently used to control the disease in Africa. However, this vaccine is considered unsafe due to adverse side effects and because it often causes viremia and has the potential of reassortment with wild-type AHSV strains, resulting in the possibility of spread of virus infection (4). Therefore, rationally designed safe AHSV vaccine candidates are currently being developed but have not been marketed yet (5–10).

AHSV is a nonenveloped virus containing 10 double-stranded RNA (dsRNA) segments (S1 to S10). The outer capsid is composed of two major structural proteins, VP2 and VP5. The serotype-determining protein VP2 is the most variable viral protein and the major target of the protective immune response. The core particle consists of two concentric layers: the surface VP7 layer and the inner VP3 layer that encloses the genome of 10 dsRNA segments and a replicase complex of three enzymes, VP1, VP4, and VP6 (11). Comparisons of the sequences of capsid proteins VP2, VP3, VP5, and VP7 of BTV serotype 10 (BTV10) with those from EHDV serotype 1 and AHSV4 have revealed the close structural relationship between these viruses: the inner core proteins, VP3 and VP7, are the most conserved, whereas the outermost proteins, VP2 and VP5, are the most variable (12). Previous structural studies of BTV have revealed the atomic-level details for BTV particle organization (13, 14), and it is expected that the AHSV architecture has a structural organization similar to that of BTV (15). However, at the level of individual proteins, only the structure of a top domain of VP7 has been reported for AHSV4 (16); therefore, a molecular understanding of the structure-function relationship of AHSV proteins is not available and structure-based rational vaccine design for AHSV is not currently possible.

In the past decade, reverse genetics (RG) technology has revolutionized the understanding of BTV replication (17, 18) and pathogenesis (19, 20) and greatly advanced the development of vaccine technologies (21, 22). The RG technology for AHSV strains has been demonstrated (23, 24) but needs significant improvement to increase the efficiency of virus recovery. A similar RG-based approach has been developed for live-attenuated AHSV4, providing a promising approach for vaccine development (7).

The RG technology for BTV, where defective VP6 (encoded by S9) was complemented by a VP6-expressing cell line, made possible the rapid generation of disabled infectious single cycle (DISC) vaccine strains (22). This strategy has been demonstrated to be efficient for different serotypes by reassortment of the two variable RNA segments that encode the outer capsid proteins VP2 and VP5 with the remaining eight RNA segments of BTV1, including the single-stranded RNA (ssRNA) that encodes the defective VP6. Importantly, DISC viruses showed efficient replication in the complemented cell line but no virus production in any other mammalian or insect cell lines. The vaccination of cattle and sheep with such DISC viruses either singly or in a cocktail followed by challenge with virulent BTV strains showed neither a clinical reaction nor any detectable viremia (25).

Based on this highly promising DISC BTV vaccine technology, we aimed to improve and adapt it for the generation of AHSV vaccine strains. First, we employed an RG system for AHSV1 for the production of high titers of viruses solely in a complemented cell line. Once we achieved the defective AHSV1 strain, we utilized this strain as a backbone to develop defective-virus vaccines specific for all nine serotypes. However, in spite of the structural similarity between BTV and AHSV, it became clear that unlike the results obtained with the BTV serotypes, the exchange of only VP2 and VP5 of the other serotypes with the respective segments of AHSV1 did not generate viable reassortant viruses, indicating that the other proteins are not highly conserved between AHSV1 and the different serotypes. It was therefore necessary to exchange additional segments for certain serotypes. These vaccine strains were then tested for their protective efficacies in type I interferon receptor (IFNAR)-knockout (IFNAR^−/−^) mice. When vaccinated mice were challenged with two virulent strains of AHSV, complete protection against homologous infection was achieved, demonstrating the suitability of the vaccine strains as vaccine candidates.

## MATERIALS AND METHODS

### Cells and viruses.

BSR cells (subclones of BHK-21 cells) were maintained in Dulbecco modified Eagle medium (DMEM; Sigma) supplemented with 5% fetal bovine serum (FBS; Invitrogen). The stable cell line BSR-VP6 was grown in DMEM–5% FBS supplemented with 7.5 μg/ml of puromycin (Sigma). Equine dermal (E. Derm) cells (NBL-6, ATCC CCL-57) were cultured in Eagle minimum essential medium (MEM; Sigma) supplemented with 10% FBS and 1% nonessential amino acids. Mammalian cell lines were cultured at 37°C in a 5% CO_2_ humidified atmosphere. Insect KC cells, derived from Culicoides midges (26), were maintained at 28°C in Schneider's insect medium supplemented with 10% FBS.

AHSV serotypes 1 to 9 were kindly supplied by S. Zientara (ANSES, France). All AHSV serotypes were passaged once in BSR cells, titrated, and used for subsequent experiments. The AHSV4 challenge strain was kindly provided by José Manuel Sánchez-Vizcaíno (Universidad Complutense de Madrid, Spain).

### Plasmids.

For the AHSV1 RG system, the coding regions of the corresponding segments were inserted in the pCAG-PM vector (18) using AflII and PacI restriction sites. The corresponding expression plasmids were designated pCAG-AHSV1VP1, pCAG-AHSV1VP3, pCAG-AHSV1VP4, pCAG-AHSV1VP6, pCAG-AHSV1VP7, pCAG-AHSV1NS1, and pCAG-AHSV1NS2 and confirmed by sequencing. T7 plasmids carrying AHSV transcripts were generated using a sequence-independent cloning system as previously described (17). Briefly, genomic dsRNA was ligated to a self-annealing primer before reverse transcription (RT)-PCR amplification with an adaptor primer. Each cDNA amplified from AHSV segments was cloned into the pUC19 vector and sequenced.

An AHSV1 mutant (S9multistop) containing 11 stop codons (TAA or TGA) throughout the S9 gene was created by gene assembly and the introduction of mutations at positions 288 to 304 (3 stop codons and an NS4 frameshift), 377 to 385 (3 stop codons), 590 to 607 (3 stop codons), and 872 to 877 (2 stop codons), and the sequence was confirmed.

### Development of the BSR-VP6 cell line constitutively expressing AHSV1 VP6.

A complemented cell line expressing AHSV1 VP6, the BSR-VP6 cell line, was generated as described previously (18) with some modifications. Briefly, BSR cells were transfected by electroporation at 240 V and 975 μF with an AHSV1 VP6-expressing vector, pCAG-AHSV1VP6. After electroporation, a suspension of cells was seeded onto 150-mm culture plates and VP6-expressing colonies were selected in the presence of 7.5 μg/ml of puromycin. Surviving clones were tested by immunoblotting analysis using polyclonal guinea pig antiserum raised against AHSV6 VP6, which was cross-reactive with AHSV1 VP6. The best-expressing clone was used for the recovery of VP6-defective viruses.

### Recovery of wild-type (wt) AHSV1 from T7 RNAs.

The T7 promoter and exact 3′ end containing DNAs were used as the templates in equimolar proportions to produce a mixture of 10 capped T7 RNA transcripts for AHSV1 using an mMESSAGE mMACHINE T7 Ultra kit (Ambion) according to the manufacturer's instructions. For the recovery of a strain by RG (the recAHSV1 strain), BSR cells at 50% confluence in 12-well plates were transfected with a set of expression plasmids carrying the genes for AHSV1 VP1, VP3, VP4, VP6, VP7, NS1, and NS2 in different combinations (80 ng of each plasmid per well). At 16 h posttransfection (hpt), cells were transfected for a second time with a total of 500 ng of all 10 capped RNA transcripts. To assess the RNA infectivity, at 4 h after the second transfection the monolayers were overlaid with 1.5% low-temperature agarose (catalog number A9045; Sigma) in DMEM containing 1% FBS and incubated for 2 to 3 days at 35°C until the formation of plaques. Alternatively, to collect recovered viruses, the transfection medium was replaced with DMEM containing 1% FBS and incubation was continued for 2 to 3 days until the appearance of a cytopathic effect.

### Recovery of def-AHSV from T7 RNAs.

For recovery of VP6/NS4-deficient AHSV1 (def-AHSV1), BSR-VP6 cells at 50% confluence in 12-well plates were transfected with 5 pCAG plasmids carrying the genes for VP1, VP3, VP4, VP6, and NS2 (80 ng each), followed by a second transfection at 16 hpt with a total of 500 ng of 10 capped AHSV1 RNA transcripts: those for S1 to S8, S10, and S9multistop. To obtain reassortant def-AHSVs, several segments were used to replace parental AHSV1. Each defective virus was plaque purified and titrated on BSR-VP6 cells. To assess stability, each defective virus was passaged at least 5 times in BSR-VP6 cells at a multiplicity of infection (MOI) of 0.1. Analysis of the dsRNA profile and RT-PCR/sequencing were used to confirm the integrity of the introduced AHSV segments.

### *In vitro* growth kinetics of AHSV.

The growth kinetics of wt AHSV and defective viruses were determined in BSR-VP6, BSR, KC, and E. Derm cells following infection for 1.5 h at MOIs of 0.1, 5, 2.5, and 10, respectively. After infection, the inoculum was removed and the cells were washed twice with medium supplemented with 1% FBS. At 0, 24, 48, and 72 h postinfection (hpi), the supernatant was harvested and the titer in BSR-VP6 cells was determined by plaque assay and expressed as the number of PFU per milliliter. Each experiment was performed in triplicate and repeated twice. Three strains of wt AHSV1 were used in this experiment: recAHSV1 (obtained by RG from exact copies of T7 RNAs), wt AHSV1 (obtained from second BSR cell passage of the original AHSV1 stock), and AHSV_KC_ (virus passaged twice in KC cells at an MOI of 0.1). All three AHSV stocks showed similar results (data not shown); therefore, data for only one strain (recAHSV1) are presented.

### Mouse vaccination.

Thirty IFNAR^−/−^ mice with a C57BL/6 mouse genetic background were obtained from the specific-pathogen-free breeding unit of the Friedrich-Loeffler Institut (Germany) and assigned to 5 groups; male and female animals were distributed equally. Six mice each were immunized subcutaneously twice 3 weeks apart with 10^6^ PFU (100 μl) of def-AHSV1 or def-AHSV4 and were also challenged by the same route 21 days after the second immunization with 10^5^ 50% tissue culture infective doses (TCID_50_) in 100 μl of either AHSV serotype 1 (the def-AHSV1-immunized group) or AHSV serotype 4 (the def-AHSV4-immunized group). Twelve mice were mock vaccinated with phosphate-buffered saline (PBS); six of them were infected with AHSV1, and six of them were infected with AHSV4. Six further mice were kept as controls. The experimental protocol was reviewed by a state ethics commission (Landesamt für Landwirtschaft, Lebensmittelsicherheit und Fischerei Mecklenburg-Vorpommern [LALLF]) and has been approved by the competent authority (reference number LALLF M-V/TSD/7221.3-1.1-058/10).

All mice were weighed daily for 10 days after vaccination and infection and examined for clinical signs, which included weight loss, rough hair, lethargy, and apathy; but no neurological signs, such as ataxia, paresis of one or more legs, or paralysis, were observed, as reported previously (27). Animals showing severe symptoms were immediately euthanized, and the remaining infected and untreated control animals were euthanized at 2 or 3 weeks after challenge. Blood samples were obtained from all mice at 3, 7, and 10 days after infection. At autopsy, blood, spleen, liver, and brain samples were taken, and the organ samples were homogenized in 1 ml serum-free MEM. RNA from 20 μl blood or 100 μl tissue homogenate was extracted using a KingFisher 96 Flex purification system (Thermo Scientific, Braunschweig, Germany) in combination with a MagAttract virus mini M48 kit (Qiagen, Hilden, Germany) according to the manufacturers' instructions and analyzed by a VP7-based real-time RT-quantitative PCR (qPCR) assay (28) combined with an internal control system targeting the beta-actin housekeeping gene (29).

## RESULTS

### Establishment of reverse genetics for AHSV.

**FIG 1:**
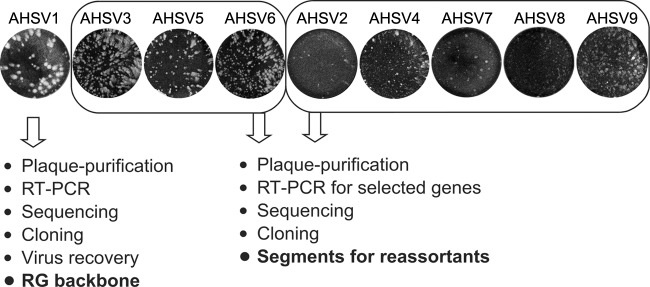
Genetic and phenotypic analysis of AHSV serotypes. The plaque morphology in BSR cells infected with the indicated AHSV serotypes and incubated for 48 h is shown. The viruses were divided into groups with large (AHSV1), medium (AHSV3, -5, and -6), and small (AHSV2, -4, -7, -8, and -9) plaque phenotypes. The strategy for developing the RG backbone virus (AHSV1) and the recovery of selected segments for the other 8 AHSV serotypes is shown below.

An efficient RG system offers a rapid approach for manipulation of the viral genome that allows the molecular mechanisms of viral genes and gene products to be understood. The previously published AHSV RG system used transfection of a mixture of two different serotypes, namely, six plasmids expressing AHSV6, followed by 10 T7 RNA transcripts of AHSV4, in order to recover viable virus (23). However, this combination was found to be insufficient for molecular manipulations due to the low efficiency of recovery of virus, which was present at titers of less than 10^6^ PFU/ml. Therefore, it was necessary to identify a well-replicating AHSV serotype with uniform large plaques for virus recovery by the RG system. All nine wt AHSV serotypes were then tested on BSR cells to assess virus titers and plaque phenotypes (Fig. 1). Three distinct groups of AHSV serotypes were observed: a large defined plaque phenotype with high titers (AHSV1), a medium plaque phenotype with high titers (AHSV3, -5, and -6), and a small/undefined plaque phenotype (AHSV2, -4, -7, -8, and -9) with low titers. Four well-replicating AHSV serotypes (AHSV1, -3, -5, and -6) were analyzed for genetic stability via sequencing of several plaque-purified isolates, and three (AHSV3, -5, and -6) showed high genetic diversity. To avoid any complication, plaque-purified AHSV1, which exhibited a large uniform plaque phenotype and high titers (5 × 10^8^ PFU/ml), was considered to be the most suitable for use in an RG system.

**FIG 2:**
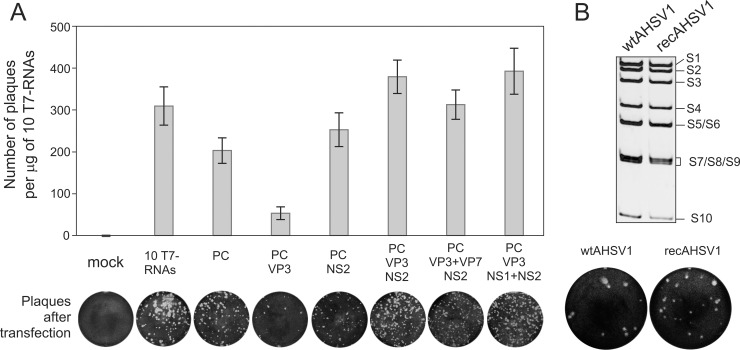
Establishment of reverse genetics system for AHSV1. (A) Minimum requirements for primary replication of AHSV1. BSR cells were transfected in triplicate with the set of indicated expression plasmids (PC, polymerase complex proteins VP1, VP4, and VP6), followed by a second transfection with 10 capped AHSV1 T7-derived transcripts (S1 to S10). (Top) The bar chart shows the number of plaques per microgram of 10 T7 RNAs; (bottom) the plaque morphology of each combination indicated above is shown. The bars represent the average ± standard deviation. (B) (Top) Purified genomic dsRNA profiles of wt AHSV1 and RG-recovered virus (recAHSV1) analyzed on a 9% nondenaturing polyacrylamide gel. The positions of the genomic dsRNA segments are indicated on the right. (Bottom) Plaque formation of wt and recAHSV1 stocks in BSR cells.

The recovery of full copies of the 10 AHSV1 RNA segments was performed by a sequence-independent method as described previously (17). For each of the 10 segments, a T7 promoter-derived plasmid with the exact 3′ end was generated to produce 10 capped T7 RNA transcripts. The protein-coding sequences of VP1, VP3, VP4, VP6, VP7, NS1, and NS2, which form the primary replicase complex, were used to generate expression plasmids under the control of the pCAG promoter (24). For the RG system, we tested several conditions to mimic the preformation of the primary replication complex. The transfection of 3 to 7 expression plasmids followed by the transfection of exact copies of 10 T7 RNA transcripts was performed. A combination of 5 expression plasmids (expressing VP1, VP3, VP4, VP6, and NS2) was found to be sufficient to generate high numbers of AHSV plaques and was used throughout the subsequent experiments (Fig. 2A). When an isolated plaque (recAHSV1) was amplified in BSR cells, virus titers were as high as 4 × 10^8^ PFU/ml, equivalent to that of the wt virus. Both the plaque morphology and genomic dsRNA profile were similar to those of the wt AHSV1 (Fig. 2B). These data demonstrate that the efficient AHSV1 RG system requires primary transfection of subcore and NS2 expression plasmids followed by transfection of 10 exact copies of the ssRNA transcripts.

### Generation of a stable cell line expressing an essential gene of AHSV1.

**FIG 3:**
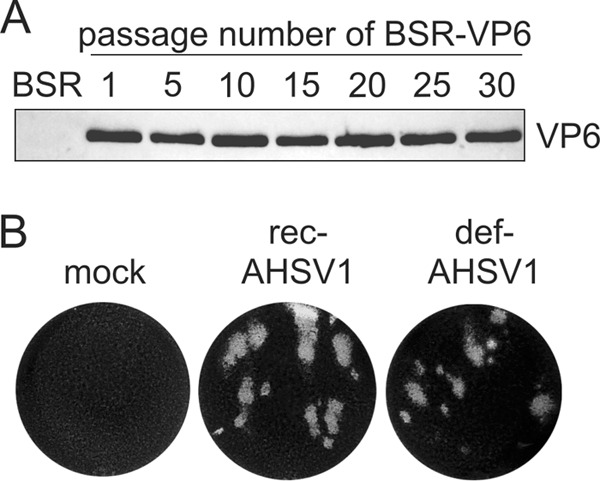
Stable cell line (BSR-VP6 cells) expressing VP6 to complement defective AHSV1. (A) The complemented cell line expressing AHSV1 VP6 was generated, and one selected clone was passaged 30 times at a 1:20 dilution. Equal numbers of cells from the indicated passages were analyzed by immunoblotting using a VP6-specific antibody. Normal BSR cells were used as a control. (B) Plaque morphology of defective virus (def-AHSV1) in BSR-VP6 cells recovered by the RG system. Mock- and recAHSV1-infected cells were used as controls under the same conditions used for def-AHSV1.

The efficient RG system should be suitable for the generation of targeted, highly defective AHSV strains, facilitating functional and structural studies without the requirement of a high-containment environment. It has previously been shown that VP6, a product of S9, is involved in both ssRNA packaging and helicase activity (30). Therefore, we decided to develop VP6-deficient viruses, which required the generation of a cell line complemented with VP6. The ability of *trans*-complementation of VP6 in the RG system of the related virus BTV has led to the development of VP6-deficient mutant viruses that are able to replicate in the complemented cell line but not in BSR or insect KC cells (18, 22). Therefore, we created a BSR cell line expressing AHSV1 VP6 (the BSR-VP6 cell line) by electroporation of BSR cells with pCAG-AHSV1VP6, followed by puromycin selection. The selected clone stably expressed VP6 for 30 passages at low dilutions (Fig. 3A).

In addition to VP6, S9 also encodes a minor nonstructural protein, NS4, which is dispensable for BTV replication in BSR cells (31). To construct VP6/NS4-deficient AHSV1 (def-AHSV1), we introduced multiple nucleotide changes in S9 of AHSV1. Instead of the use of deletions reported for VP6-deficient BTVs (22), we introduced 11 stop codons (TAA or TGA) throughout the S9 gene (S9multistop), disrupting the open reading frames (ORFs) of VP6 and NS4 but retaining the length of the S9 RNA, thus minimizing the genetic pressure on defective AHSV1 recovery and replication. The absence of VP6 in def-AHSV1 was expected to be compensated for by the complemented cell line.

**FIG 4:**
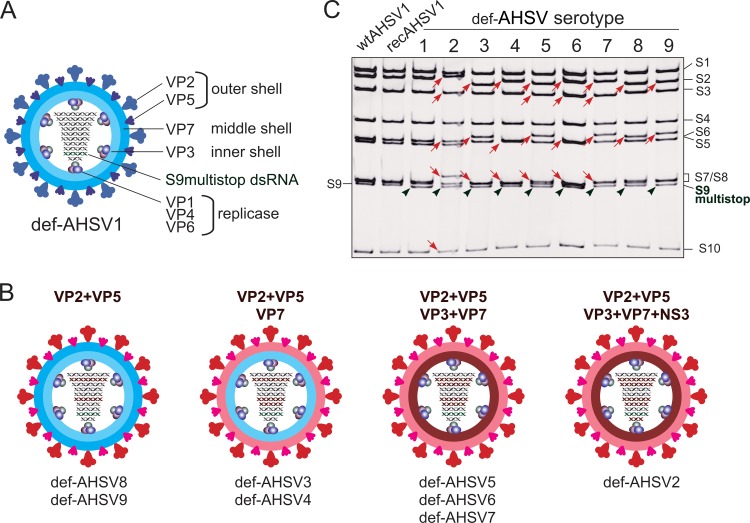
Reassortant-defective AHSV variants. (A) Schematic representation of def-AHSV1 backbone defective virus depicting the S9multistop segment. (B) Schematic representation of reassortant-defective viruses AHSV2 to AHSV9. The segments exchanged in order to achieve high titers and a large plaque phenotype in the BSR-VP6 cell line are indicated on top. The cartoons of defective AHSV particles illustrate the origins of four major structural proteins to be AHSV1 (blue) or another serotype (red). The red dsRNA segments indicate the exchanged segment (S2, S3, S6, S7, and S10). The serotypes of each reassortment group obtained are indicated below. (C) The pattern of genomic dsRNA purified from the BSR-VP6 cell line infected with wt AHSV1, recAHSV1, and defective AHSV variants (5th passage at an MOI of 0.1 on the BSR-VP6 cell line) was analyzed on a 9% nondenaturing polyacrylamide gel. The positions of the genomic dsRNA segments are indicated on the right. The wt S9 dsRNA position is indicated on the left, whereas the S9multistop dsRNA of defective AHSV variants is indicated by green arrowheads. The red arrows on the gel correspond to the exchanged segments used for the recovery of defective AHSV variants.

Using the newly developed BSR-VP6 complemented cell line, def-AHSV1 could be recovered by transfection of 5 expression plasmids followed by transfection of 10 capped T7 RNA transcripts, where S9 was replaced by the S9multistop segment. After the second transfection, agar-overlaid monolayers were used to assess RNA infectivity, which was comparable to that of wt AHSV1 (Fig. 3B). Recovered virus was plaque purified using the BSR-VP6 cell line and amplified once titers of 1 × 10^8^ PFU/ml (titrated on the complemented BSR-VP6 cell line) were reached. The plaque morphology (Fig. 3B) and analysis of genomic dsRNA segments showed the expected dsRNA profile (Fig. 4C), confirming that AHSV VP6 can be efficiently complemented and NS4 is dispensable for AHSV replication in BSR cells.

### Efficient assembly of heterologous AHSV particles requires reassortment of several segments.

The ability of orbiviruses to reassort provides the possibility to coat the conserved replicase complex or inner core with the outer capsid, thereby resulting in serotype-specific particles. Such particles may have broad application spectra, for example, as vaccine candidates or safe platforms for structural and molecular studies. It has previously been demonstrated that the exchange of AHSV VP2 only can lower the reassortant virus titers up to 3 log units (7), resulting in impaired replication and/or particle instability. Therefore, we aimed to utilize def-AHSV1 as a backbone to achieve well-replicating defective strains of all 8 other AHSV serotypes (AHSV2 to AHSV9) by exchanging combinations of different segments.

Initially, exact copies of segments S2 (VP2), S6 (VP5), S7 (VP7), S3 (VP3), and S10 (NS3/NS3A) of each serotype were cloned and sequenced. Amino acid sequence analysis of the major structural proteins of the nine AHSV serotypes revealed the increases in the degree of conservation in the expected order: VP2 was the least conserved (identity, 13 to 71%; similarity, 27 to 84%), followed by VP5 (identity, 75 to 99%; similarity, 90 to 99%) and then VP7 and VP3, which were the most conserved (identity, >98%; similarity, >99%). To produce T7 RNA transcripts, exact copies of T7 DNA clones of genomic segments were generated as described above.

For defective-virus recovery, BSR-VP6 cells were transfected with the complete set of T7 RNAs of AHSV1, where S9 was replaced by S9multistop and, depending on the serotype, the combinations S2-S6, S2-S6-S7, S2-S6-S7-S3, or S2-S6-S7-S3-S10 were used to replace the equivalent segments of def-AHSV1. The criteria for the selection of defective viruses were the plaque size (clear plaques after 2 to 3 days of incubation at 35°C) and the final titer of the plaque-purified viruses determined using the BSR-VP6 cell line (10^7^ to 10^8^ PFU/ml). These requirements were chosen to minimize any possible pressure to avoid recombination and instability of the modified dsRNA genome. As expected, the exchange of only S2 (VP2) resulted in either no virus recovery (def-AHSV2, -3, and -5) or very poor recovery with titers in the range of 2 × 10^3^ to 5 × 10^5^ (def-AHSV4, -6, -7, -8, and -9). These observations are consistent with those for previously published AHSV reassortants based on S2 exchange (7). To develop stable and high-titer defective viruses of the different serotypes, replacement of two outer capsid proteins with def-AHSV1 was not sufficient for all serotypes. While well-replicating def-AHSV8 and def-AHSV9 could be achieved by the reassortment with only 2 segments (S2 and S6) encoding the outer shell proteins VP2 and VP5, def-AHSV3 and def-AHSV4 were achieved only when, in addition to S2 and S6, the homologous S7, encoding the scaffolding VP7 protein for deposition of VP2 and VP5, was also exchanged (Table 1). Three additional serotypes were created by reassortment with 4 segments (S2, S6, S7, S3), resulting in def-AHSV5, def-AHSV6, and def-AHSV7. This set was created by complete replacement of the major structural protein shell. Further, to generate well-replicating (in BSR-VP6 cells) reassortant def-AHSV2, five segments (S2, S6, S7, S3, S10) had to be replaced to achieve high titers on the complemented BSR-VP6 cell line (Table 1). In each case, when these required combinations of segments were not replaced, defective viruses (AHSV2, 3-, -4, -5, -6, and -7) were poorly recovered and these viruses exhibited a small plaque phenotype and low titers, ranging from 10^4^ to 10^6^ PFU/ml. Therefore, these were excluded from the subsequent studies. The schematic representation of all selected defective viruses is summarized in Fig. 4A and B. All 9 defective viruses were passaged 5 times at an MOI of 0.1 in the complementing BSR-VP6 cell line, confirming at each step that none of the tested stocks was able to replicate on wild-type BSR cells. To assess the stability and integrity of reassortant-defective viruses, dsRNA was extracted from stocks of the initial virus recovery and the final (fifth) passage. Each dsRNA sample exhibited the correct mobility (Fig. 4C). Further, RT-PCR and sequencing also validated the authenticity of the RNA segments in all defective-virus strains.

**TABLE 1:** RNA segment requirements for the recovery of nine defective AHSV serotypes

def-AHSV serotype	Exchange of heterologous segment:					Virus titer (PFU/ml)
	S2 (VP2)	S3 (VP3)	S6 (VP5)	S7 (VP7)	S10 (NS3)	
def-AHSV1^*a*^						2 × 10^7^ to 9 × 10^7^
def-AHSV2	+	+	+	+	+	1 × 10^7^ to 4 × 10^7^
def-AHSV3	+		+	+		5 × 10^7^ to 7 × 10^7^
def-AHSV4	+		+	+		3 × 10^7^ to 1 × 10^8^
def-AHSV5	+	+	+	+		2 × 10^7^ to 3 × 10^7^
def-AHSV6	+	+	+	+		2 × 10^7^ to 3 × 10^7^
def-AHSV7	+	+	+	+		2 × 10^7^ to 3 × 10^7^
def-AHSV8	+		+			8 × 10^7^ to 1 × 10^8^
def-AHSV9	+		+			7 × 10^7^ to 1 × 10^8^

a def-AHSV1 is the parental strain.

### Defective AHSVs do not grow in AHSV-susceptible cell lines but yield high titers in the complemented cell line.

**FIG 5:**
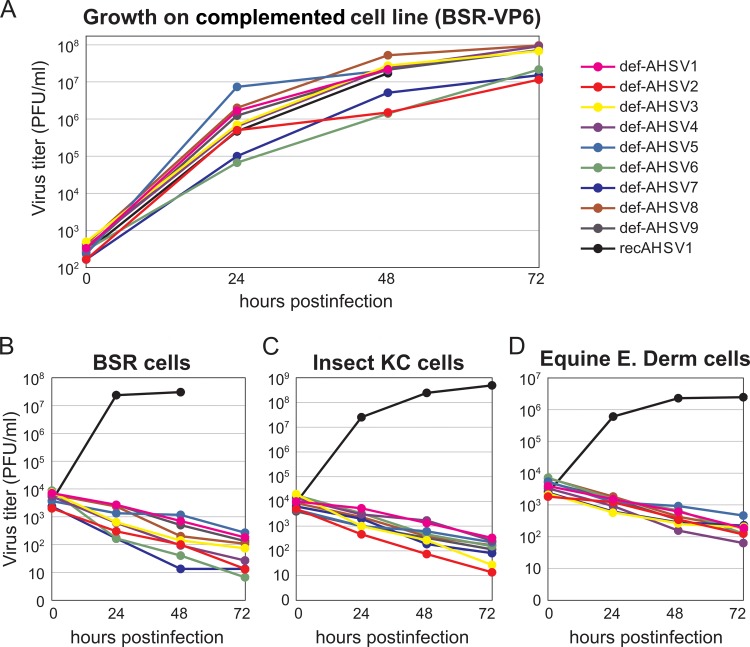
Growth of VP6-deficient viruses in mammalian and insect cell lines. (A) Virus growth kinetics in the complemented BSR-VP6 cell line. BSR-VP6 cells were infected with the indicated viruses at an MOI of 0.1. The titer was analyzed by plaque assay on BSR-VP6 cells at 0, 24, 48, and 72 hpi. (B to D) Virus growth kinetics in normal BSR (B), insect KC (C), and E. Derm (D) cells. The three cell lines were infected at an MOI of 5 (BSR cells), 2.5 (KC cells), and 10 (E. Derm cells). The titer was analyzed by plaque assay on BSR-VP6 cells at 0, 24, 48, and 72 hpi.

To determine the growth kinetics of each of the defective viruses, complemented BSR-VP6 cells were infected with each virus at a low MOI, and the virus titers were determined at 24, 48, and 72 hpi. Similar to wt AHSV1, all 9 defective viruses were capable of replication in the complemented BSR-VP6 cells, reaching titers in the range of 10^7^ to 10^8^ PFU/ml and forming plaques at 2 to 3 days postinfection (Fig. 5A). Each def-AHSV variant was propagated five times at a low MOI in complemented cells (BSR-VP6 cells). Aliquots of each passage corresponding to 5 × 10^7^ to 1.5 × 10^8^ particles were then tested in wt BSR cells to ensure the defective property. As expected, there was no virus growth, confirming the safety of the def-AHSV variants.

To confirm that both parental def-AHSV1 and the eight reassortant-defective viruses were incapable of growth in normal cells, the growth kinetics of each defective virus were evaluated by infecting three different normal cell lines (BSR, Culicoides KC cells, and E. Derm cells) at a high MOI and measuring the virus titers at 24, 48, and 72 hpi. The results demonstrated that these defective viruses (def-AHSV1 to def-AHSV9) were not able to grow in any of these three cell lines (Fig. 5B to D). To ensure that there was no delayed replication, all infected cells were incubated for an additional 7 days; however, no signs of infection were detected (data not shown). When the results are taken together, we conclude that all nine defective viruses grew to high titers (between 3 × 10^7^ and 1 × 10^8^ PFU/ml) in the complemented cell line (Table 1) but did not produce viable virions in normal BSR, KC, and E. Derm cells. Considering that the standard vaccination dose per horse is 10^6^ to 10^7^ virus particles, for a quadrivalent vaccine the maximum injected volume will not exceed 0.5 to 1 ml, since 2 × 10^7^ cells infected with each def-AHSV serotype should provide at least ∼75 to 200 doses, suggesting that such productivity is appropriate for clinical applications and mass production. These results demonstrate a great potential for these defective viruses to be used as safe AHSV vaccine candidates.

### Defective AHSV1 and AHSV4 efficiently protect IFNAR^−/−^ mice from homologous infection.

**FIG 6:**
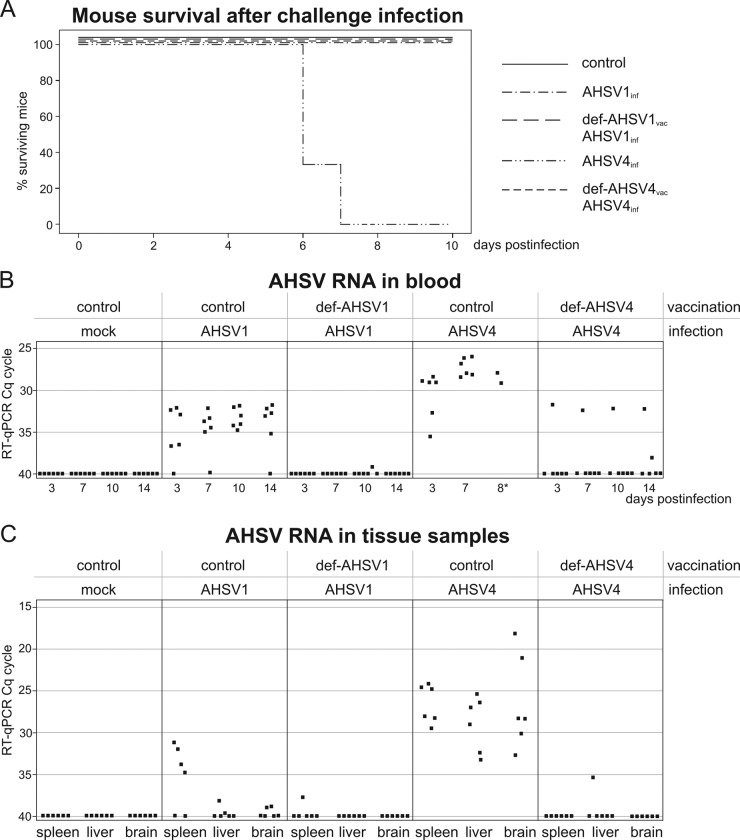
Protection of IFNAR^−/−^ mice by def-AHSV1 and def-AHSV4 vaccination from homologous challenge infection. (A) Survival plots of adult IFNAR^−/−^ mice (6 per group) immunized with 10^6^ PFU of def-AHSV1 or def-AHSV4 (i.e., vaccinated [vac]) and challenged (i.e., infected [inf]) with 10^5^ TCID_50_ of the homologous wt AHSV1 or wt AHSV4 strain. (B and C) RNAemia in blood (B) and organs (spleen, liver, brain) (C) of the indicated groups of animals is presented as a graph of inverted *C_T_* values obtained by RT-qPCR. *C_T_* values higher than 40 were considered negative. Each dot corresponds to one analyzed animal sample. Cq, quantification cycle.

As a proof of principle, def-AHSV1 and reassortant def-AHSV4 were assessed for their protective efficacy against AHSV infection in an animal model. Since adult interferon-knockout (IFNAR^−/−^) mice had been shown to be susceptible to AHSV (27) and proved to be an appropriate model with which to test for AHSV vaccine efficacy in horses (10), two groups of mice were immunized twice with 10^6^ PFU of either def-AHSV1 or def-AHSV4. No adverse side effects were observed after vaccination, and the body weights of immunized and control animals showed no significant differences (a maximum 10% variation was observed for all groups). At 3 weeks after the second immunization, vaccinated and control animals were challenged subcutaneously with virulent AHSV1 or AHSV4. Protection indicators included body weight measurements, survival times, and blood and tissue AHSV-specific RNA levels. The group of 6 control mice, which were not vaccinated but which were challenged with AHSV4, started to lose weight from day 3 or 4 postchallenge onwards. Four animals were euthanized at day 7 after infection (because of severe weight loss, rough hair, and apathetic behavior), and the two remaining mice were euthanized on day 8. The nonvaccinated mouse group infected with AHSV1, representing a less virulent serotype, showed no clinical signs. Also, groups of mice vaccinated with either def-AHSV1 or def-AHSV4 showed no clinical signs (Fig. 6A).

The levels of viremia, determined by analysis of the amount of AHSV-specific RNA present in blood, were measured in all mice at 3, 7, and 10 days after infection. No AHSV-specific RNA was detected in any mock-infected mice. Mice infected with AHSV4 had significantly higher AHSV RNA levels than mice infected with AHSV1 (Fig. 6B). These data correlated with the mouse survival times (Fig. 6A), suggesting that AHSV1 represents a less virulent serotype. In immunized animal groups, the levels of viremia were significantly reduced (Fig. 6B). The analysis of AHSV-specific RNA was combined with an internal control system targeting the beta-actin housekeeping gene, which was present in all collected blood samples (threshold cycle [*C_T_*] values, 26 to 32).

Spleen, liver, and brain tissue samples were taken for RNA extraction, and RNA was quantified as described in Materials and Methods. Consistent with the previous data (Fig. 6A and B), high loads of AHSV RNA were measured in all organs of nonvaccinated mice with AHSV4 infection, whereas the analyzed organs of the AHSV1-infected group demonstrated from low AHSV RNA levels to no AHSV RNA. As expected, vaccination with def-AHSV1 and def-AHSV4 prior to homologous AHSV challenge led to a significant reduction of AHSV RNA levels in mouse organs (Fig. 6C). Nonimmunized mice infected with AHSV1 and AHSV4 showed detectable levels of AHSV-specific RNA, but AHSV1-infected mice had no mortality or weight loss, in contrast to the findings for AHSV4-infected mice. For this reason, the AHSV-specific RNA level was chosen as the main protection indicator for analysis of the potency of the vaccine candidates.

## DISCUSSION

African horse sickness remains an actual threat to the species of equidae in Africa and countries surrounding the Mediterranean. Although live-attenuated polyvalent vaccines protecting against almost all AHSV serotypes, except serotypes 5 and 9, are commercially available, these vaccine strains have a high risk of reassortment with field isolates, which results in the introduction of new virus serotypes into countries where AHSV is not endemic (32). Therefore, in the last 2 decades, various attempts have been made to develop safe and efficacious AHSV vaccines; however, to date none of these has been developed into a commercial product. For successful vaccine preparation, in addition to safety and efficacy, it is essential that some other criteria also be met, including technical simplicity and reproducibility, ease of scaling up of production, downstream processing of the manufactured vaccine, and most importantly, cost-effective vaccine manufacture, particularly for animal vaccines. Moreover, vaccines that could allow the differentiation of infected from vaccinated animals (DIVA) are also highly desirable. Although subunit vaccines based on purified proteins are compliant with the need for DIVA and safe, they generally do not provide sufficient and long-lasting protection and their production is costly. Orbivirus-like particles are safe and provide sufficient protection but require complex preparation methods and suffer from low yields and problems with reproducibility. Although attenuated virus-based chimeras created by the exchange of outer capsid protein VP2 alone or in combination with VP5 have good immunological potential, so far they have demonstrated significant variability in virus titers (7), which are often as low as 10^5^ to 10^6^ PFU/ml, which precludes their further development into a cost-efficient product. Therefore, in this study, we prioritized the virus productivity parameters to overcome these deficiencies for the generation of AHSV vaccine candidates.

Based on previous research on orbiviruses, we considered the creation of replication-deficient virus particles, which enclose an efficient replicase of a backbone virus coated with immunogenic capsid proteins from all AHSV serotypes, to be the most promising strategy. For this purpose, the most efficiently replicating plaque-purified AHSV1 variant was first selected as it provided the highest titers and had a defined large plaque phenotype. Second, the genome segments of this AHSV1 variant were subcloned and sequenced, and an efficient RG system, along with optimized virus recovery parameters, was established. It was found that the double-transfection approach, in which cells were first transfected with DNA expression vectors, which provided high-level expression of the primary replication complex components of 5 proteins, followed by transfection with *in vitro*-cotranscribed RNAs representing exact copies of the virus segments, was the most efficient method for AHSV recovery, similar to that previously reported for BTV (33). To create replication-defective virus, we targeted disruption of the ORF of the essential replicative enzyme VP6, as well as the overlapping ORF of the less well-studied NS4 protein, in the coding region of segment S9 of AHSV1 by introducing multiple stop codons. Notably, mutations introduced into the S9multistop segment should be sufficient to allow differentiation of infected from vaccinated animals by quantitative RT-PCR; this, however, needs to be confirmed in future experiments. To support the replication of a defective virus, it was necessary to establish a complemented BSR cell line expressing functional VP6. These cells were highly efficient in supporting the propagation of defective AHSV1. Therefore, AHSV1 was considered suitable as a backbone for the generation of defective virus strains for the remaining eight serotypes (AHSV2 to AHSV9) by exchanging the RNA segments encoding serotype-determining capsid proteins of the other serotypes. Similar strategies to create replication-deficient virus strains have been previously reported for BTV (22, 25), adenovirus (34), and herpes simplex virus and other viruses (reviewed in reference 39). Such defective viruses have an advantage as vaccines because their initial infection site mimics replicating virus, thus providing a strong and specific immune response.

Since during viral infection the majority of a neutralizing immune response is based on the VP2 protein, it would be logical to assume that exchange of the corresponding segment would be sufficient. However, since the productivity of a vaccine strains is also critical, we sought to optimize conditions to generate vaccine strains. We found that for eight AHSV serotypes (AHSV2 to AHSV9) the rescue of corresponding viruses using an established RG system was possible for some serotypes but that the titers of the viral stocks were unacceptably lower (below 5 × 10^5^ PFU/ml) than those of the parental virus. Such a low titer of the rescued virus not only would impede its further development into a cost-efficient vaccine candidate but also is indicative of the problems associated with virus assembly, dissemination, and, possibly, genetic stability. It can be expected that swapping the large, most variable RNA segment, segment S2, to alter the serotype of the virus particle may have a number of consequences. The orbivirus particle is made up of three concentric layers of proteins, whereas VP2 makes contacts with the outer capsid protein VP5 and the inner capsid protein VP7. Therefore, amino acid changes in the replaced VP2 may disturb its interactions with other capsid proteins, thus influencing the stability of the assembled virion. Additionally, orbiviral RNA segments not only need to interact with each other (36) but also may interact with the packaging protein during assembly, so the integrity of the native S2 can be crucial. Therefore, variations at the nucleotide sequence level may also result in a reduced efficiency of core assembly for heterologous virus. To this end, current knowledge about nucleotide-level requirements for RNA packaging and atomic-level structural details about AHSV virion organization is clearly lagging behind, precluding the rational design and optimization of reassortant virus particles. Therefore, we used a semirational approach by replacing S2 (VP2) along with S5 (VP5), S2 with S5 and S7 (VP7), and S2-S5-S7 with S3 (VP3) or S2-S5-S7-S3 and S10, as S10 is also highly variable among different AHSV serotypes. We assumed that the advantage of the replacement of S10 can be 2-fold, because S10 RNA has been reported to be especially important during RNA packaging (36), whereas its ORF encodes the NS3 protein, which is implicated in the virus budding process (35). By testing various combinations, we selected virus variants that efficiently replicated to titers equivalent to those of the wt virus. Interestingly, the number of segments needed to be exchanged in the backbone virus differed for different serotypes, with the extreme case being AHSV2, which required the exchange of five segments, including S10 (NS3). These data are in agreement with previously published reports on BTV, where interaction between VP2 and NS3 has been shown to be important for efficient virus egress (37). However, the possibility that RNA-RNA interactions are involved in the efficiency of reassortment between AHSV serotypes should also be considered.

Since adult IFNAR^−/−^ mice infected by AHSV show clinical disease and pathogenesis and have successfully been used for AHSV vaccine efficacy studies and the same vaccines have also conferred protection in horses, IFNAR^−/−^ mice are an appropriate model for the evaluation of the protective efficacy of the defective viruses (10, 27). When two different defective viruses, including one reassortant-defective virus, were assessed, both vaccinated groups of mice demonstrated significantly decreased levels of viremia, in contrast to the control nonvaccinated groups, which showed high levels of viremia and/or clinical signs. Although these data are highly encouraging, further vaccination/challenge experiments with different combinations of serotypes will need to be assessed using not only IFNAR^−/−^ mice but also horses.

The live-attenuated AHSV vaccines have been extensively used in South Africa for several decades. More recently, one of the LAV strains, AHSV4LP, which has a deletion of NS3/NS3A, was employed as a potential vaccine development platform (7). However, a recent report on another RNA virus has demonstrated the reversion to virulence of an attenuated severe acute respiratory syndrome coronavirus lacking E protein after it was propagated in mice (38). Therefore, the *in vitro* complementation of a missing protein would represent a safer AHSV vaccine platform with a minimized genetic pressure toward the reversion to virulence. Notably, in this study, we avoided deletion of the genes but disrupted the ORF of AHSV S9, coding for VP6 and NS4, by introducing multiple stop codons. Multiple passages of the vaccine strains in a complemented cell line did not reveal any changes in the mutated S9, strongly suggesting that the mutated ORF is unlikely to revert. Also, our results have demonstrated that all nine defective AHSVs were able to replicate in the complemented cell line to titers similar to those of the wt AHSV serotypes. Most importantly, defective viruses were unable to propagate in normal cells, whether they were of mammalian or insect origin. Unlike live-attenuated vaccine strains, the def-AHSV vaccine candidates are replication deficient in normal cells, and their activity is limited to the primary expression only of viral proteins, making the risks of recombination with circulating field strains minimal. As a result, such vaccines provide a safer option for horses than the current live-attenuated virus vaccines and have equivalent or improved protective efficiency.
